# Folate metabolism–based risk stratification identifies CYP27B1 as a determinant of tumor progression in HNSCC

**DOI:** 10.3389/fmed.2026.1814665

**Published:** 2026-05-05

**Authors:** Kai Zhang, Bingya Liu, Haixia Hu

**Affiliations:** 1 Department of General Surgery, Shanghai Institute of Digestive Surgery, Shanghai Key Laboratory of Gastric Neoplasms, Ruijin Hospital, Shanghai Jiao Tong University School of Medicine, Shanghai, China; 2 Department of Otolaryngology & Head and Neck Surgery, Ruijin Hospital, Shanghai Jiao Tong University School of Medicine, Shanghai, China

**Keywords:** chemotherapy, folate metabolism, HNSCC, prognostic biomarker, tumor microenvironment

## Abstract

Head and neck squamous cell carcinoma (HNSCC) is characterized by frequent recurrence and poor survival, highlighting the need for reliable biomarkers for risk stratification and therapeutic guidance. Folate-mediated one-carbon metabolism has been linked to tumor development; however, its prognostic and immunological relevance in HNSCC remains unclear. Using The Cancer Genome Atlas (TCGA) transcriptomic data, we developed and validated a folate metabolism–related gene signature (FMRG_score). An 11-gene model constructed by least absolute shrinkage and selection operator (LASSO) and multivariate Cox regression effectively stratified patients into distinct survival groups and served as an independent prognostic factor. A nomogram integrating FMRG_score with clinical variables demonstrated favorable predictive performance. Functional enrichment analyses revealed activation of oncogenic pathways in high-risk tumors. Immune deconvolution indicated that elevated FMRG_score was associated with reduced anti-tumor immune infiltration, increased macrophage-related signals, and an immunosuppressive tumor microenvironment. Drug response prediction suggested higher estimated IC_50_ values in the high-risk group, showing potential chemoresistance. Mechanistically, CYP27B1 knockdown suppressed malignant phenotypes and enhanced cisplatin sensitivity. Collectively, these findings establish folate metabolism as a metabolic–immune axis in HNSCC and suggest that the FMRG_score may serve as a prognostic and therapeutic stratification tool.

## 1 Introduction

Head and neck squamous cell carcinoma (HNSCC) is a common malignancy worldwide and is associated with high recurrence and poor long-term survival, despite advances in surgery, radiotherapy, chemotherapy, and immunotherapy (1, 2). Tumor heterogeneity and therapeutic resistance remain major obstacles to improving patient outcomes, underscoring the need to identify reliable prognostic biomarkers and key molecular drivers to support precision treatment strategies (3, 4).

Cancer cells frequently undergo metabolic alterations that support sustained proliferation, enhance stress tolerance, and facilitate immune evasion (5–7). Among metabolic pathways, Folate related metabolism is crucial for nucleotide synthesis (8), methylation reactions (9), redox homeostasis (10), and amino acid metabolism (11). Beyond its canonical function in supporting DNA synthesis (12), accumulating evidence suggests that folate metabolism intersects with oncogenic signaling pathways, including mTOR (13), Wnt (14), and KRAS signaling (15), thereby contributing to tumor progression and response to therapy.

In addition to promoting tumor cell proliferation, one-carbon metabolism has been implicated in shaping the tumor microenvironment (16–18). Folate-dependent metabolic flux can regulate epigenetic modifications and cytokine expression, potentially affecting immune cell recruitment, differentiation, and function (19–22). However, the systemic relationship between folate metabolism–related genes (FMRGs), immune landscape remodeling, and chemotherapeutic sensitivity in HNSCC remains poorly defined.

Although antifolate agents and nucleotide synthesis inhibitors are widely used in cancer therapy, most studies have focused on individual enzymes rather than integrated metabolic signatures (23, 24). A comprehensive evaluation of folate metabolism–associated gene networks in the context of prognosis, immune infiltration, and treatment response is still lacking. Moreover, the mechanistic roles of individual FMRGs in tumor progression and therapeutic resistance remain to be clarified.

In this study, we developed and validated a folate metabolism–related prognostic signature (FMRG_score) for HNSCC based on transcriptomic data. We then analyzed its associations with clinicopathological characteristics, immune infiltration, tumor microenvironment features, and chemotherapy response. In addition, we performed functional experiments to examine CYP27B1, a key gene in the signature, and demonstrated its role in promoting tumor growth, invasion, and cisplatin resistance *in vitro* and *in vivo*.

This study clarifies the involvement of folate metabolism as an integrated metabolic–immune regulatory axis and indicate its value in prognosis prediction and therapeutic intervention in HNSCC.

## 2 Materials and methods

### 2.1 Data collection

Transcriptomic profiles and corresponding clinical data for 566 HNSCC cases were accessed through the TCGA repository. GSE65858 dataset was used for external validation. A curated set of 616 genes related to folate metabolism was retrieved from MSigDB v2023.2. Hs. Expression values originally provided in FPKM units were rescaled to TPM and subsequently log2-transformed [log2 (TPM + 1)] prior to downstream analyses. Somatic mutation profiles, copy number variation data, and tumor mutation burden metrics were also extracted from TCGA to support integrated genomic evaluation.

### 2.2 Development and assessment of the folate metabolism–related prognostic model

Differential expression analyses comparing tumor versus normal tissues, as well as high- and low-risk groups, were performed with the “limma” package in R. Genes meeting the thresholds of |log2 fold change| > 1.3 and adjusted *p* < 0.05 were retained for further evaluation. Associations between gene expression and overall survival (OS) were examined through univariate Cox proportional hazards modeling. Candidates demonstrating prognostic relevance were subsequently subjected to LASSO regression with 10-fold cross-validation for feature selection. A composite risk score was then derived by integrating the selected genes with their corresponding regression coefficients, as defined below.

Risk Score=Σ(Expi×coefi)

Within the risk model, expression values reflect normalized transcript levels for each gene, while the associated coefficients were derived from LASSO regression. Risk stratification was performed according to the median score. Clinical variables, including age, sex, and TNM stage, were obtained from TCGA records. The independent prognostic contribution of the risk score was evaluated through Cox proportional hazards modeling.

### 2.3 Association between risk groupings and clinical characteristics

Clinical characteristics such as age, sex, and TNM stage were included in the analysis. OS between different groups was compared using Kaplan–Meier curves generated in R with the “survival” and “survminer” packages.

### 2.4 Construction and evaluation of the nomogram

To examine whether the FMRG_score retained prognostic relevance after adjustment for clinical variables, univariate and multivariate Cox models were fitted. The association between the FMRG_score and clinicopathological characteristics was further examined to assess its clinical relevance. To enhance the practical applicability and prognostic precision of the model, we developed a nomogram incorporating the FMRG_score with independently significant clinical variables, including T stage and N stage, to estimate 1-, 3-, and 5-year OS probabilities. The predictive accuracy of the FMRG_score was subsequently compared with conventional clinicopathological parameters with time-dependent ROC analysis, and predictive accuracy was assessed by AUC.

### 2.5 Functional enrichment analysis

Functional annotation of the DEGs was performed using GO and KEGG enrichment approaches together with GSEA. All computations were carried out in R (version 4.3.1) with the “clusterProfiler,” “org.Hs.eg.db,” and “enrichplot,” packages. Results meeting an adjusted FDR below 0.05 were considered statistically significant.

### 2.6 Tumor microenvironment analysis

Stromal and immune components of the tumor microenvironment were estimated using the ESTIMATE framework. Immune cell distributions were inferred through CIBERSORT and further characterized with the TIP platform.

### 2.7 Mutation and drug susceptibility analyses

Mutation profiles were processed in R with the “maftools” package, and tumor mutation burden was calculated for each case. Chemotherapy response was inferred by predicting IC_50_ values for commonly used agents through the “pRRophetic” algorithm.

### 2.8 Cell lines and culture

HNSCC cell lines CAL27 and HN30 were purchased from ATCC (United States). Cell authentication was confirmed by STR profiling. Cells were cultured in DMEM supplemented with 10% FBS, penicillin (100 U/mL), and streptomycin (100 μg/mL) at 37 °C in 5% CO₂.

### 2.9 Lentiviral-mediated knockdown of CYP27B1

To establish stable CYP27B1 knockdown cell lines, shRNA targeting CYP27B1 (5′-AGGAAGGGTGAAGCCTTATTT-3′) was cloned into the pGreenPuro vector. Lentiviral infection was performed in CAL27 and HN30 cells in the presence of 10 μg/mL polybrene in the viral supernatant. After overnight incubation, the medium was replaced with fresh complete medium. Puromycin (10 μg/mL) was added 48 h later for selection to obtain stable knockdown cell lines (CAL27/shCYP27B1 and HN30/shCYP27B1).

### 2.10 Western blotting

Proteins were isolated from cultured cells in RIPA lysis buffer (Kangwei, Beijing, China) containing phosphatase inhibitors (Roche), and concentrations were measured using the BCA method (Pierce, United States). Equal protein quantities (20 μg) were subjected to electrophoresis and subsequently transferred onto 0.22 μm PVDF membranes (Millipore, United States). After blocking in 5% milk solution, membranes were incubated overnight at 4 °C with primary antibodies targeting CYP27B1 (Abcam, ab206655) and GAPDH (Proteintech). HRP-conjugated secondary antibodies (Thermo Fisher Scientific) were applied, and immunoreactive bands were visualized using ECL detection reagents (Thermo Fisher Scientific) with the Tanon 5,200 system (Tanon, China).

### 2.11 CCK8 assays

Proliferative capacity was determined using the CCK-8 method (Dojindo, Japan). Cells were distributed into 96-well plates at a density of 2 × 10^3^ per well. After incubation with CCK-8 reagent for 2 h at 37 °C, absorbance was measured at 450 nm using a BioTek reader. Data were normalized to the first-day OD value, and relative growth was calculated for later time points. Experiments were repeated three times independently.

### 2.12 Transwell assays

Transwell assay were performed as previously described (25).

### 2.13 Wound-healing assays

Wound-healing assay were performed as previously described (25).

### 2.14 *In vivo* tumorigenicity assay

BALB/c nude male mice aged 4–6 weeks (SPF (Beijing) Biotechnology Co., Ltd., China) were housed under pathogen-free conditions at Ruijin Hospital’s animal facility. Animal use followed institutional ethical standards. Groups consisted of five mice each. CAL27/shCYP27B1 cells (2 × 10^6^) in 150 μL PBS were administered subcutaneously. Tumor growth was monitored every 4 days with caliper measurements, and tumor volume was estimated as L × W^2^/2. Blinded assessment was applied during measurement. Four weeks after cell implantation, mice were anesthetized and sacrificed, and tumors were excised and weighed.

### 2.15 Ethics approval statement

Participation in the study required written informed consent, which included permission to publish identifiable data or images when applicable. Animal procedures were approved by the Laboratory Animal Ethics Committee of Ruijin Hospital (Approval No. SYXK2023-0038) and carried out under established institutional guidelines.

### 2.16 Statistical analysis

Statistical analyses were carried out using R (version 4.3.3) and GraphPad Prism (version 10.0.1). Comparisons between groups were performed using Student’s t-test or the chi-square test when appropriate. Spearman’s correlation was used to assess associations between variables. Survival analysis was conducted using Cox regression models. The concordance index (C-index) was calculated to evaluate the predictive ability of the model. A two-sided *p* value < 0.05 was considered statistically significant.

## 3 Result

### 3.1 Establishment and assessment of the FMRG_score model

**Figure 1:**
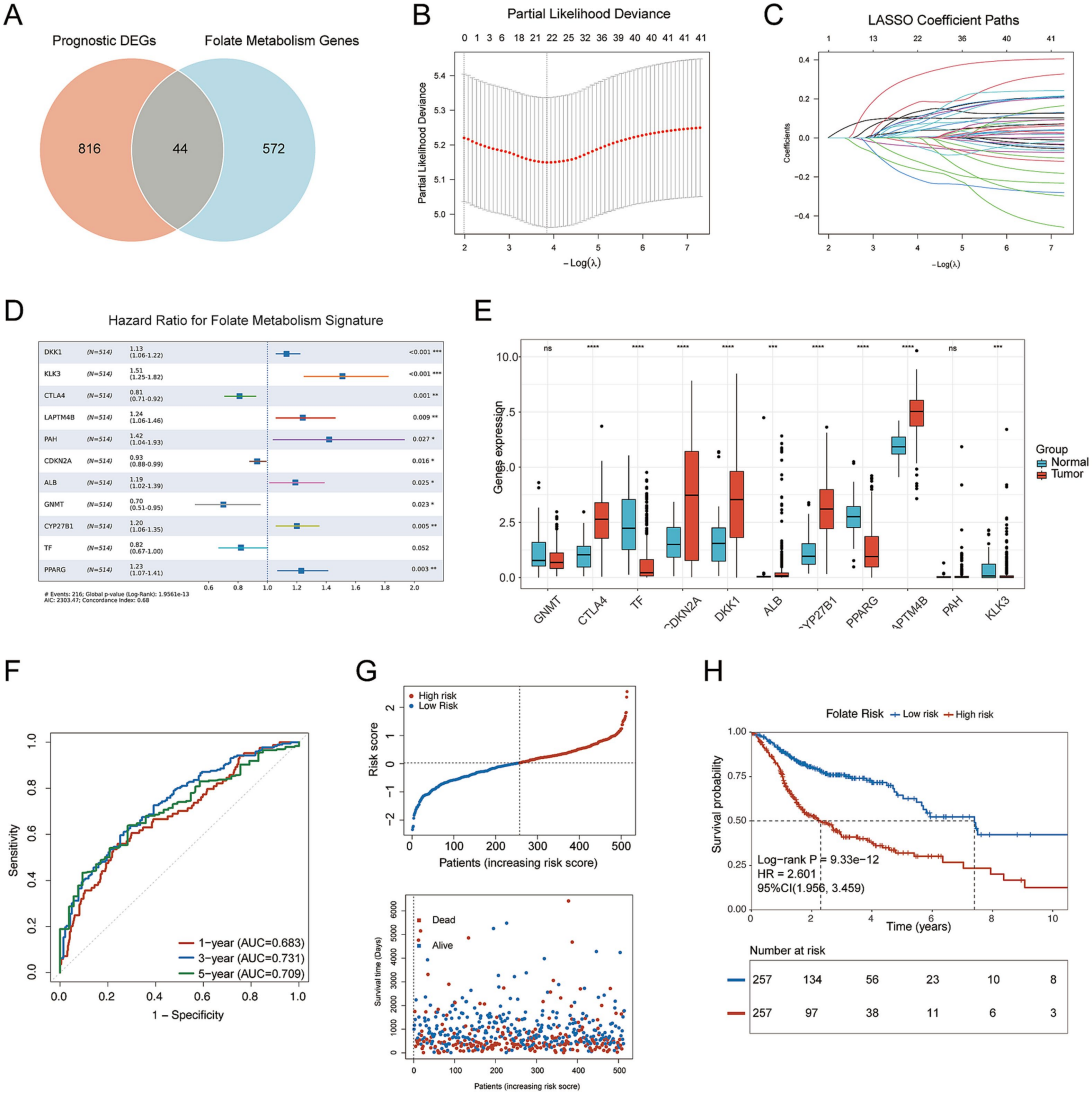
Construction of a prognostic model based on folate metabolism-related genes (FMRGs) model in HNSCC. **(A)** Veen plots of folate metabolism-related genes and prognostic DEGs in TCGA-HNSCC. **(B,C)** LASSO coefficient profiles **(B)** and partial likelihood deviance plot **(C)** for gene selection. **(D)** Identification of independent prognostic genes by multivariate Cox regression. **(E)** Boxplot comparing the expression levels of key genes in normal vs. tumor tissues. **(F)** Time-dependent ROC curves at 1, 3, and 5 years illustrating the predictive performance of the FDRG-based risk model in the TCGA cohort. **(G)** Risk scores, survival status (blue for deceased, red for alive), and scatterplot of patients based on risk score **(H)** Kaplan–Meier survival curves comparing OS between high-risk and low-risk groups. Statistical significance is marked as ****p < 0.0001, ***p < 0.001, **p < 0.01, *p < 0.05, ns, not significant.

To identify prognostic folate metabolism–related genes, univariate Cox regression and differential expression analyses were conducted in the TCGA cohort (Supplementary Figure S1A), resulting in 860 prognostic DEGs. Intersecting these genes with 616 folate metabolism–associated genes from MSigDB database yielded 44 candidate genes (Figure 1A). The univariate Cox regression results of these 44 genes are presented in (Supplementary Figure S1B). These genes were subsequently subjected to LASSO Cox regression analysis to construct a prognostic signature. The optimal penalty parameter (*λ*) was determined based on the minimum partial likelihood deviance (Figure 1B), and the corresponding coefficient profiles are presented (Figure 1C). Ultimately, 22 genes were identified. Analysis of the TCGA-HNSCC dataset demonstrated that DKK1, CTLA4, LAPTM4B, TGFBI, CDKN2A, MAP1, and CYP27B1 were markedly upregulated in tumor tissues, while KLK3, TF, GNMT, TFRC, and PPARG were significantly downregulated relative to normal tissues (Supplementary Figures S1C,D).

The genes selected by LASSO Cox regression were further subjected to multivariate Cox regression analysis to establish the final folate metabolism–related prognostic model (Figure 1D). Ultimately, We incorporated 11 genes into the final prognostic model. In the multivariate model, DKK1, KLK3, LAPTM4B, PAH, CYP27B1, and PPARG were independently associated with poorer survival (HR > 1), while CTLA4, CDKN2A, GNMT, and others were associated with favorable prognosis (HR < 1). The C-index of the FMRG_score model was 0.68, indicating moderate prognostic discrimination. The signature’s performance was validated in the external cohort GSE65858 (Supplementary Figure S2). Each patient’s risk score was calculated using the following formula:

$$\begin{array}{l} \text{Risk score}=(0.1250)\times \mathrm{DKK1}+(0.4112)\times \\ \mathrm{KLK3}+(-0.2123)\times \mathrm{CTLA4}+\\ (0.2149)\times \text{LAPTM}4B+(0.3502)\times \\ \mathrm{PAH}+(-0.0705)\times \text{CDKN}2A+\\ (0.1764)\times \mathrm{ALB}+(-0.3578)\times \text{GNMT}+\\ (0.1786)\times \mathrm{CYP}2\mathrm{7B1}+(-0.2030)\times \mathrm{TF}+\\ (0.2073)\times \text{PPARG} \end{array}$$

Comparative transcriptomic analysis revealed distinct gene expression patterns between tumor and normal samples (Figure 1E), consistent with a role in disease progression. Predictive accuracy was evaluated using time-dependent ROC analysis, with AUCs of 0.683, 0.731, and 0.709 at 1, 3, and 5 years (Figure 1F). Risk stratification based on the median score separated patients into high- and low-risk groups (Figure 1G). Mortality events were more frequent among individuals with elevated scores. Survival curves further showed significantly poorer OS in the high-risk group (log-rank *p* = 9.33e−12; HR = 2.601, 95% CI: 1.956–3.459; Figure 1H).

Collectively, these findings indicate that the folate metabolism–related gene signature serves as a robust prognostic predictor and effectively stratifies patients into distinct survival risk groups.

### 3.2 Association between clinical features and the FMRG-based signature

**Figure 2:**
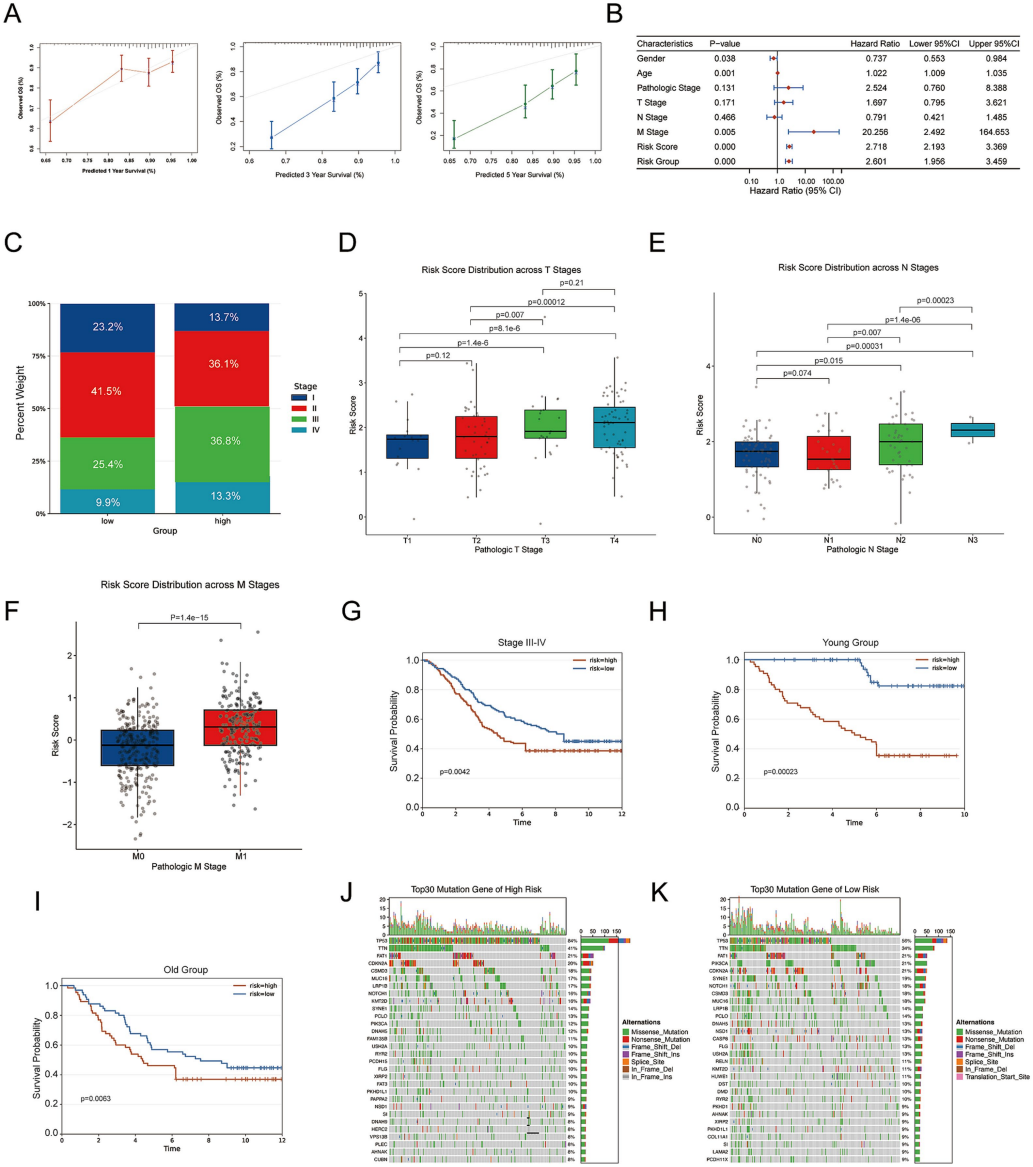
Clinical relevance and prognostic value of the FMRGs-based risk signature in the TCGA cohort. **(A)** Calibration curves of the nomogram demonstrating good concordance between predicted and observed OS at 1, 3, and 5 years. **(B)** Forest plots from univariate Cox regression analyses demonstrating that the risk score is an independent predictor of OS. **(C–F)** Boxplots illustrating the distribution of risk scores across different clinical subgroups, including pathological stage (I–IV), T stage (T1–T4), N stage (N0–N2), and M stage (M0–M1), in the TCGA cohort. **(G–J)** Kaplan–Meier analyses of OS stratified by pathological stage and age demonstrated that patients classified as high risk had significantly worse survival across all subgroups. **(J,K)** Oncoprint analysis showing the distribution of frequently mutated genes in the high-risk and low-risk subgroup.

To estimate survival probabilities, a nomogram incorporating FMRG_score and clinical factors was generated (Supplementary Figure S3A). Agreement between predicted and observed outcomes was supported by calibration curves (Figure 2A). Cox regression modeling confirmed that the risk score remained prognostically relevant after accounting for other clinical variables, together with M stage (Figure 2B). Higher scores corresponded to poorer survival outcomes. Associations with clinicopathological parameters revealed enrichment of advanced-stage tumors within the high-risk group, while early-stage cases were more common among low-risk patients (Figure 2C). In addition, risk scores increased in parallel with higher T, N, and M classifications (Figures 2D–F), reflecting more aggressive disease.

Subgroup analyses confirmed consistent survival differences across clinical classifications. Among patients with advanced-stage disease, those assigned to the high-risk category displayed significantly shorter OS (Figure 2G). The same trend was detected in age-stratified analyses, where high-risk status remained associated with poorer outcomes in both younger and older subsets (Figures 2H,I). Comparison of genomic profiles demonstrated distinct mutational landscapes between risk groups (Figures 2J,K). The high-risk category showed enrichment of mutations in several key oncogenic drivers, which may underlie its more aggressive clinical course.

Taken together, the risk score correlated with disease progression, stage, and genomic heterogeneity, and independently predicted patient outcomes.

### 3.3 Biological features linked to the FMRG-related signature

**Figure 3:**
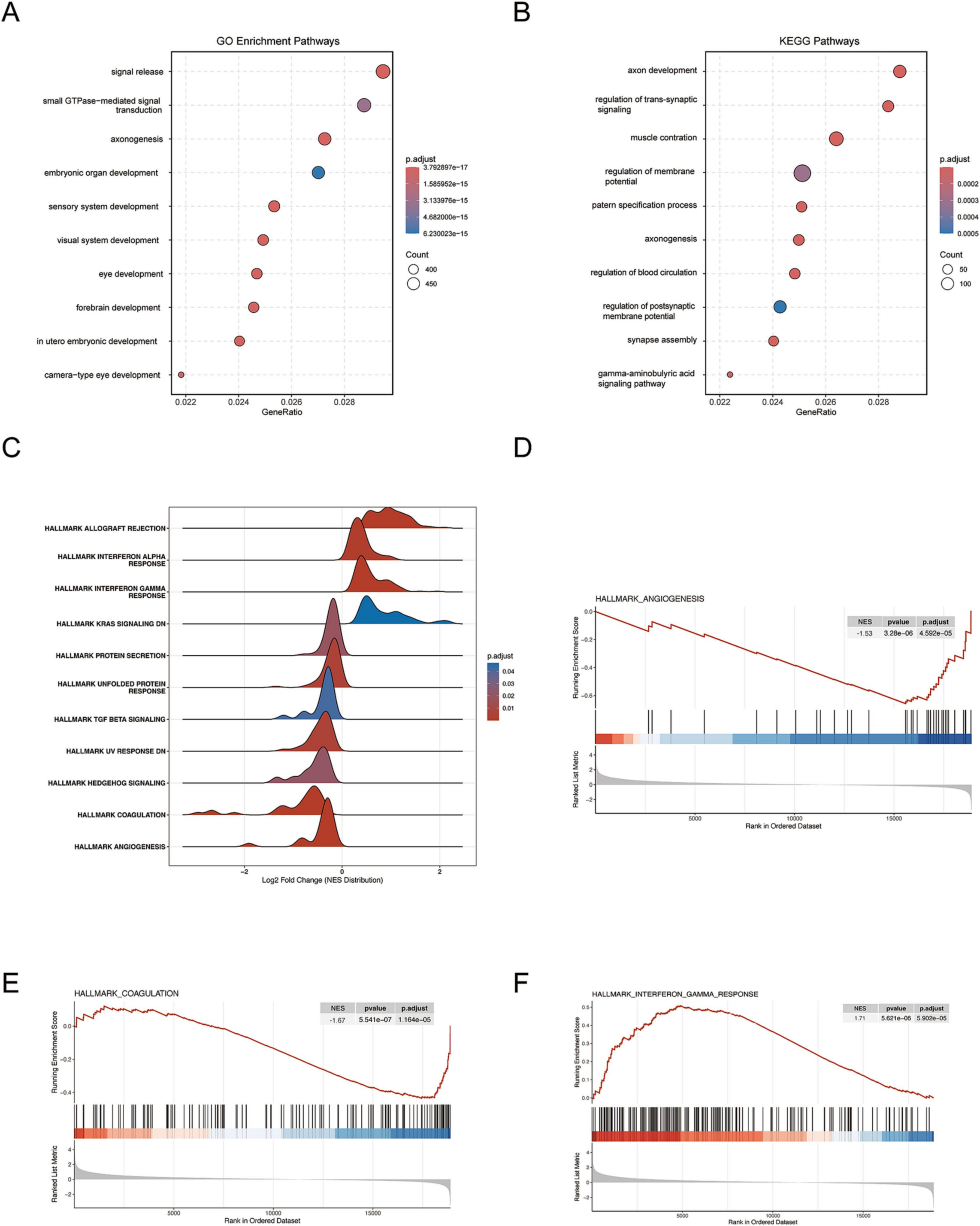
Biological characterization of the FMRGs-related prognostic signature. **(A)** Bar plot of Gene Ontology (GO) enrichment analysis comparing biological processes between the high- and low-risk groups. **(B)** Bar plot of KEGG pathway enrichment analysis comparing functional pathways between the high- and low-risk groups. **(C–F)** Gene Set Enrichment Analysis (GSEA) plots for key biological processes.

To explore the biological context of the risk model, enrichment profiling was carried out. GO-based analysis demonstrated significant representation of genes associated with signal release, small GTPase-mediated pathways, axonogenesis, and sensory development among those differentially expressed between risk categories (Figure 3A). KEGG analysis identified enrichment in pathways related to axon development, regulation of trans-synaptic signaling, membrane potential regulation, muscle contraction, and synapse assembly (Figure 3B). These results suggest that pathways associated with neural signaling may be involved in the high-risk group. GSEA indicated significant enrichment of the following pathways in the high-risk group: several Hallmark pathways, including angiogenesis, coagulation, interferon-*γ* response, interferon-*α* response, TGF-β signaling, KRAS signaling, unfolded protein response, and allograft rejection (Figures 3C–F). Enrichment plots confirmed increased activation of HALLMARK_ANGIOGENESIS, HALLMARK_COAGULATION, and HALLMARK_ INTERFERON_GAMMA_RESPONSE in the high-risk group.

Overall, tumors in the high-risk category displayed increased angiogenesis, altered immune regulation, and activation of oncogenic pathways, features consistent with a more aggressive clinical course.

### 3.4 Immune landscape and tumor microenvironment features of the risk signature

**Figure 4:**
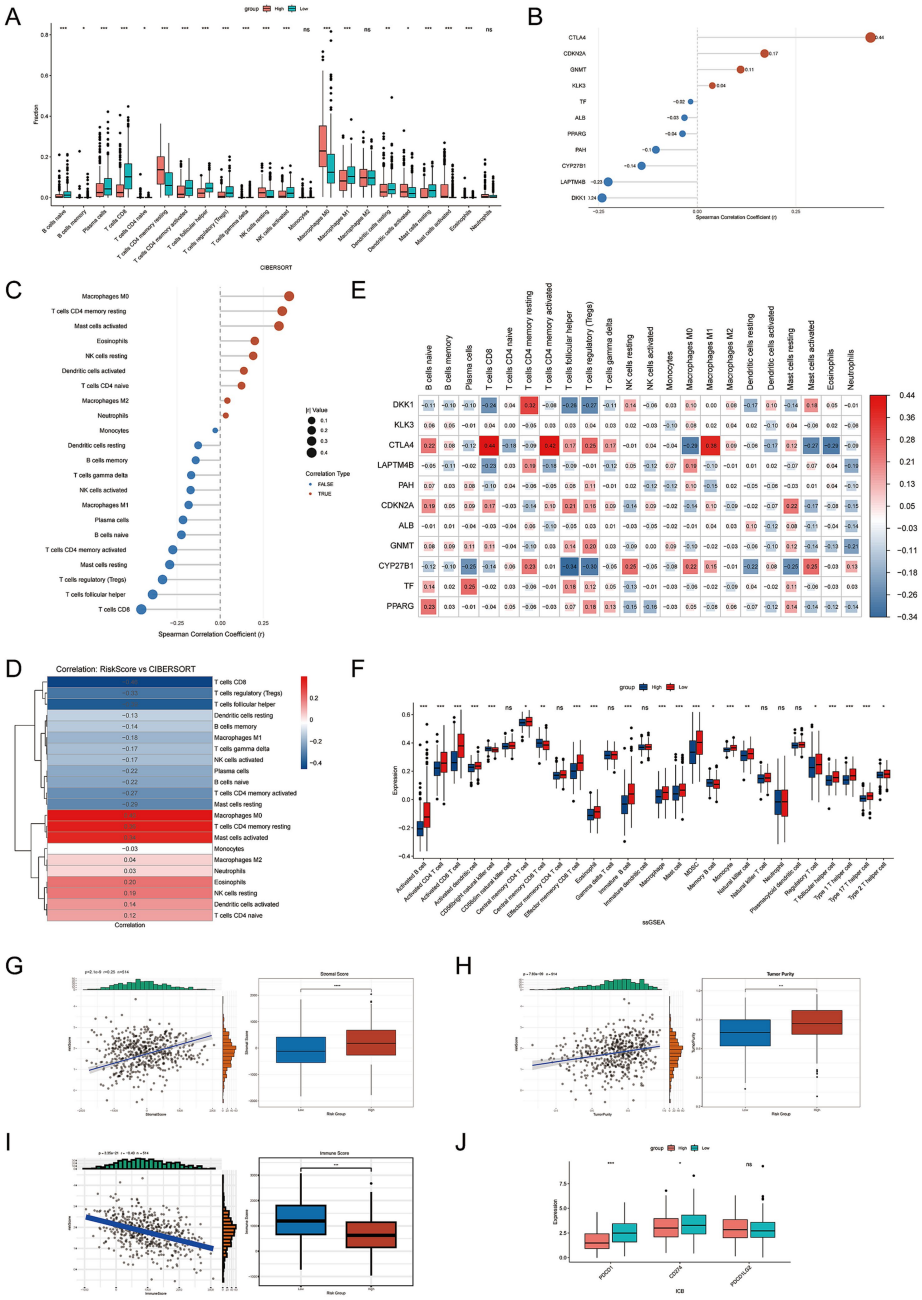
Correlation between risk score and tumor microenvironment (TME) signatures in HNSCC. **(A)** Boxplots comparing the proportions of CIBERSORT-estimated immune cell subsets between high- and low-risk groups. **(B)** Correlation between individual FMRGs and overall immune infiltration levels. **(C)** Spearman correlation analysis between the FMRGs-based risk score and infiltrating immune cell fractions estimated by CIBERSORT. Red dots represent positive correlations, and blue dots represent negative correlations. Dot size indicates correlation strength. **(D)** Heatmap summarizing the correlations between risk score and CIBERSORT immune cell fractions. **(E)** Heatmap showing the correlation coefficients between individual FMRGs and CIBERSORT-inferred immune cell subsets. Color intensity reflects the magnitude of Spearman correlation coefficients. **(F)** ssGSEA analysis comparing immune-related functional signatures between high- and low-risk groups. **(G)** Correlation between risk score and stromal score calculated using the ESTIMATE algorithm. **(H)** Association between risk score and tumor purity estimated by ESTIMATE. **(I)** Correlation between risk score and immune score. **(J)** Expression levels of immune checkpoint molecules (e.g., PDCD1, CTLA4, and CD274) in high- and low-risk groups. Statistical significance is marked as ****p < 0.0001, ***p < 0.001, **p < 0.01, *p < 0.05, ns, not significant.

To characterize immune variation across risk strata, immune cell composition was evaluated using multiple deconvolution approaches. Distinct differences in cellular distribution were observed between groups (Figure 4A). Tumors with elevated risk scores were marked by increased proportions of macrophages M0 and resting CD4 memory T cells, whereas tumors with lower scores contained greater fractions of CD8^+^ T cells and regulatory T cells. Gene-level assessment identified heterogeneous relationships between individual FMRGs and immune subsets (Figures 4B,C). The heatmap overview (Figure 4E) highlighted variable associations across macrophage and T-cell populations. Several genes exhibited notable links to macrophage subsets and adaptive immune components. When considering the composite score, higher values coincided with reduced abundance of CD8^+^ T cells, Tregs, follicular helper T cells, and activated memory CD4 T cells. In contrast, macrophages M0 and resting CD4 memory T cells became more prominent as the score increased (Figure 4D). Comparable patterns were detected with xCell and ssGSEA analyses (Supplementary Figure S4A; Figure 4F), which supported consistent shifts in immune-related pathways and cellular distributions between risk categories. Evaluation of the broader tumor microenvironment using ESTIMATE indicated that increasing risk scores were accompanied by greater stromal content and higher tumor purity, alongside reduced immune infiltration (Figures 4G–I). Overall, tumors in the high-score category exhibited a microenvironment characterized by expanded stromal components and diminished immune presence. Assessment of immune checkpoint expression further identified differences in PDCD1, CD27, and PDCD1LG2 between risk groups (Figure 4J).

Taken together, variation in the FMRG signature corresponded with differences in immune cell distribution, microenvironment composition, and checkpoint molecule expression, implying a role in modulating immune dynamics and therapy sensitivity in HNSCC.

### 3.5 Association between the FMRG signature and chemotherapeutic sensitivity

**Figure 5:**
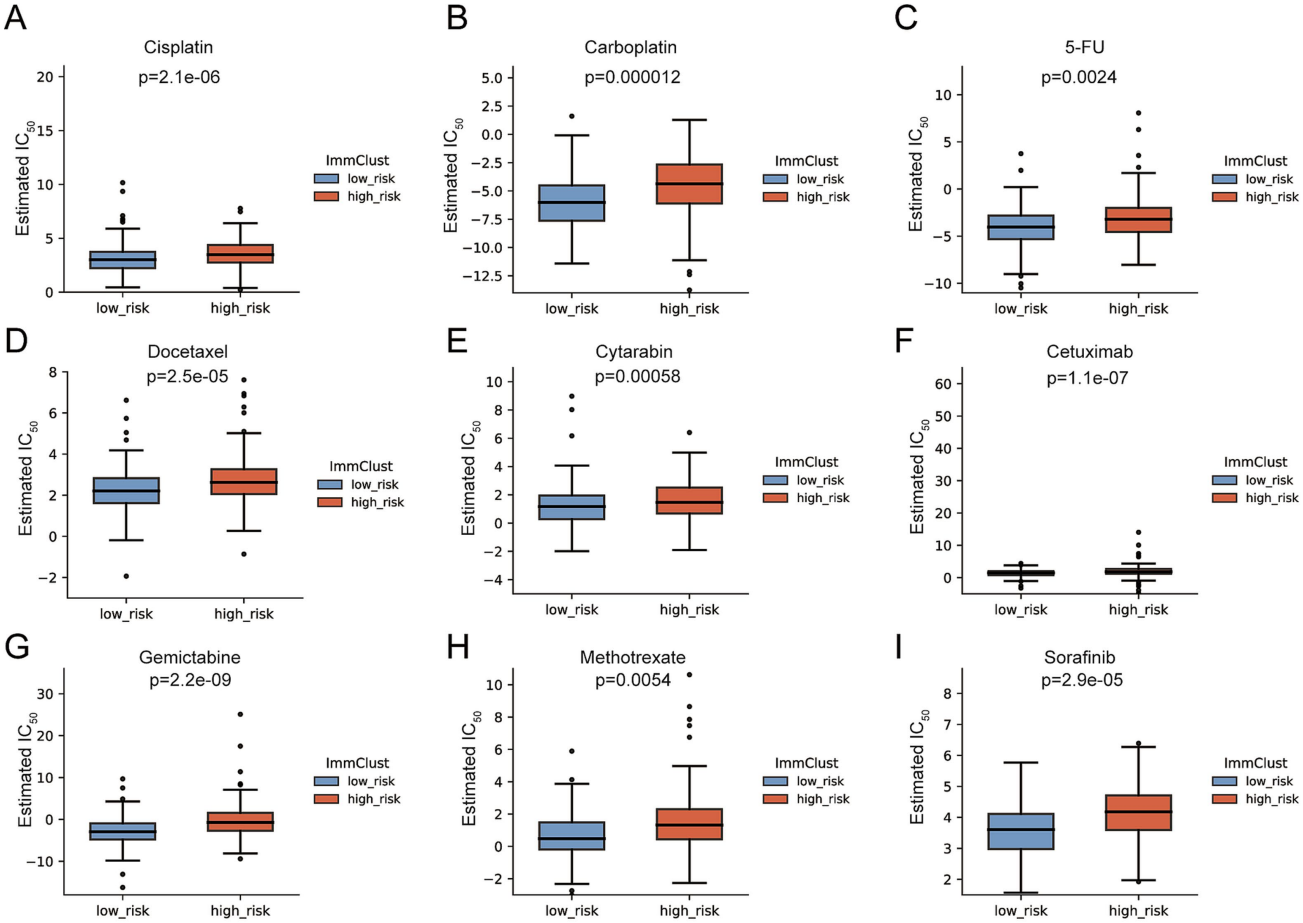
Predicted chemotherapy and targeted therapy sensitivity in high- and low-risk groups. **(A–I)** Boxplots showing the estimated IC_50_ values of multiple chemotherapeutic and targeted agents in low- and high-risk groups. Compared with the low-risk group, the high-risk group exhibited significantly higher estimated IC_50_ values for Cisplatin **(A)**, Carboplatin **(B)**, 5-Fluorouracil **(C)**, Docetaxel **(D)**, Cytarabine **(E)**, Cetuximab **(F)**, Gemcitabine **(G)**, Methotrexate **(H)**, and Sorafenib **(I)**. These findings suggest reduced drug sensitivity and increased therapeutic resistance in the high-risk group.

Predicted drug response differed between risk categories (Figure 5). Tumors classified as low risk were associated with reduced estimated IC_50_ values for cisplatin (*p* = 2.1e−06) and gemcitabine (*p* = 2.2e−09), indicating enhanced susceptibility to these agents. A comparable trend was noted across additional drugs, including carboplatin, 5-fluorouracil, docetaxel, cytarabine, cetuximab, methotrexate, and sorafenib, where higher IC_50_ estimates were consistently observed in the high-risk group. These results indicate that a higher FMRG score is associated with reduced response to chemotherapy, whereas patients with lower scores may benefit more from standard treatment. Folate-dependent pathways contribute to cellular one-carbon flux, supporting nucleotide production, epigenetic regulation, and maintenance of redox homeostasis. Several commonly used chemotherapeutic agents, such as 5-fluorouracil and gemcitabine, target pathways related to nucleotide synthesis. In addition, folate-related pathways may interact with signaling networks that regulate cell growth and survival.

Overall, variation in the FMRG score was linked to differences in predicted drug response, indicating its potential relevance for therapeutic selection. Additional clinical validation will be required to determine its applicability in practice.

### 3.6 HNSCC cells with higher CYP27B1 activity display accelerated growth, greater migratory and invasive potential, and diminished responsiveness to cisplatin

**Figure 6:**
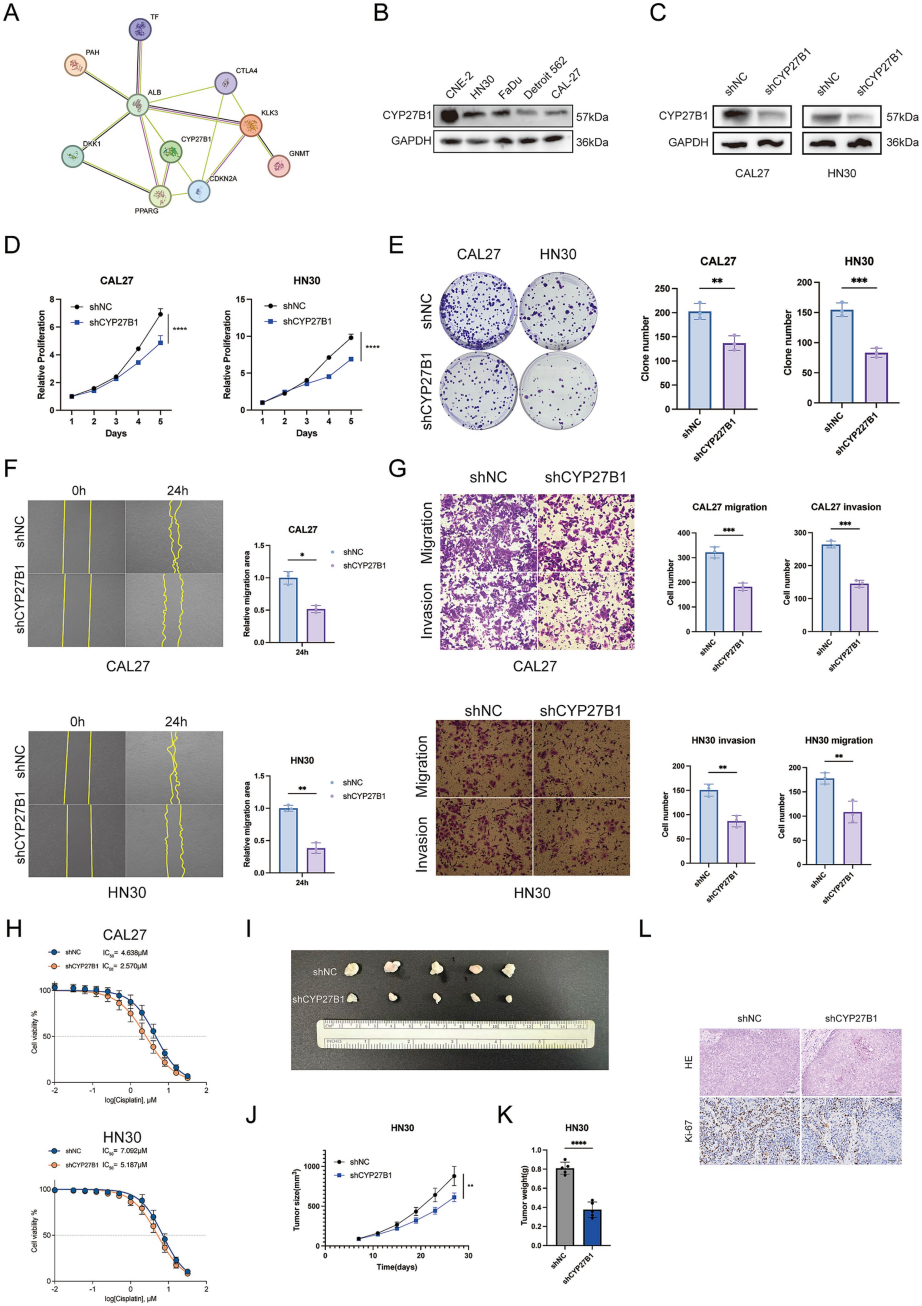
CYP27B1 promotes proliferation, migration, invasion and cisplatin resistance in HNSCC *in vitro* and *in vivo*. **(A)** Protein–protein interaction (PPI) network of CYP27B1 and its interacting partners generated from database analysis, suggesting potential involvement in tumor-related signaling pathways. **(B)** Western blot analysis of CYP27B1 protein expression in HNSCC cell lines (CNE-2, HN30, FaDu, Detroit 562 and CAL-27). **(C)** Validation of CYP27B1 knockdown efficiency in CAL27 and HN30 cells using shRNA (shCYP27B1). GAPDH was used as a loading control. **(D)** Cell proliferation assays showing that CYP27B1 knockdown significantly suppresses cell growth in CAL27 and HN30 cells compared with shNC controls. **(E)** Colony formation assays demonstrating that silencing CYP27B1 markedly reduces clonogenic capacity in CAL27 and HN30 cells. Representative images (left) and quantitative analysis (right) are shown. **(F)** Wound healing assays showing that CYP27B1 depletion significantly inhibits migratory ability of CAL27 **(F)** and HN30 **(H)** cells at 24 h. **(G)** Transwell migration and invasion assays demonstrating that CYP27B1 knockdown reduces the migratory and invasive capacity of CAL27 **(G)** and HN30 **(I)** cells. Representative images (left) and quantitative analyses (right) are shown. **(H)** Cisplatin dose–response curves in CAL27 and HN30 cells. CYP27B1 knockdown significantly decreases the IC_50_ value of cisplatin, indicating enhanced chemosensitivity. **(I)** Representative images of xenograft tumors derived from shNC and shCYP27B1 HN30 cells. **(J,K)** Tumor growth curves and final tumor weights of xenografts showing that CYP27B1 silencing significantly suppresses in vivo tumor growth. **(L)** Hematoxylin and eosin (H&E) staining and Ki-67 immunohistochemical staining of xenograft tumor tissues. CYP27B1 knockdown reduces Ki-67 expression, indicating decreased proliferative activity (Scale bars: 50 μm). Data are presented as mean ± SD. *p < 0.05, **p < 0.01, ***p < 0.001.

Protein interaction relationships within the FMRG signature were examined using STRING, revealing that CYP27B1 occupies a central position with multiple strong connections. (Figure 6A). Western blot analysis confirmed that CYP27B1 was expressed in several HNSCC cell lines (Figure 6B). Given the relatively high endogenous expression of CYP27B1 in CAL27 and HN30 cells, these cell lines were transduced with lentiviral vectors carrying shCYP27B1 to establish stable knockdown cell lines. Successful suppression of CYP27B1 expression was confirmed by Western blot analysis (Figure 6C). Knockdown of CYP27B1 reduced cell proliferation in both CAL27 and HN30 cells (Figure 6D). Colony formation was also decreased after CYP27B1 silencing (Figure 6E). In wound healing assays, cells with CYP27B1 knockdown showed slower migration (Figure 6F). Transwell assays further confirmed reduced migration and invasion in both cell lines (Figure 6G). These results suggest that CYP27B1 promotes the malignant behavior of HNSCC cells. Given the predicted association between FMRGs and chemotherapy sensitivity, we next assessed the impact of CYP27B1 on cisplatin responsiveness. Dose–response analysis showed that CYP27B1 knockdown reduced the IC_50_ of cisplatin in both CAL27 and HN30 cells. (Figures 6H,I), suggesting enhanced chemosensitivity upon CYP27B1 suppression. To validate these findings *in vivo*, xenograft experiments were performed. Tumors derived from shCYP27B1 cells were markedly smaller than those from control cells (Figures 6J,K). Tumor growth curves confirmed significantly reduced tumor volume and weight following CYP27B1 silencing. Histological examination showed decreased Ki-67 staining in the shCYP27B1 group (Figure 6L), indicating reduced proliferative activity in vivo.

Collectively, these results demonstrate that CYP27B1 promotes tumor growth, invasion, and cisplatin resistance in HNSCC, highlighting its potential as a therapeutic target.

## 4 Discussion

In the present study, we systematically established and validated a folate metabolism–related gene signature (FMRG_score) that robustly stratifies HNSCC patients into distinct prognostic groups. By integrating transcriptomic profiling, survival modeling, immune landscape analysis, therapeutic response prediction, and functional validation, Our results show that altered folate metabolism is associated with tumor progression, changes in the immune microenvironment, and chemotherapy resistance.

Folate metabolism is essential for nucleotide synthesis and one-carbon metabolism; however, accumulating evidence suggests that its impact extends far beyond these canonical functions (26–28). Our prognostic model, derived from 11 independent genes selected through LASSO and multivariate Cox regression analyses, maintained good predictive ability at different time points and remained an independent prognostic factor after adjusting for clinical variables. In addition, the FMRG_score was associated with advanced TNM stage and increased metastasis, suggesting a link between folate metabolism and tumor aggressiveness.

These findings align with emerging studies suggesting that metabolic reprogramming is not merely a byproduct of tumor growth but a driver of malignant progression. One-carbon metabolism, in particular, supports DNA methylation, redox balance, and biosynthetic flux, thereby influencing genomic stability and tumor evolution (21, 29–31).

Functional enrichment analyses showed that high-risk tumors were enriched in angiogenesis, KRAS signaling, TGF-β signaling, unfolded protein response, and coagulation pathways. These pathways are closely linked to tumor proliferation, stress adaptation, and metastatic dissemination (32–36). Interestingly, enrichment of neural-related biological processes and synaptic signaling pathways suggests a potential interaction between metabolic remodeling and tumor–nerve crosstalk, an emerging hallmark of tumor progression (37). The observed enrichment of angiogenic and inflammatory pathways in high-risk tumors further supports the notion that folate metabolic dysregulation contributes to a pro-tumorigenic microenvironment. Such integration of metabolic and signaling pathways may explain the strong association between FMRG_score and clinical outcomes.

Immune characteristics varied across FMRG_score levels. Tumors with elevated scores were characterized by reduced proportions of CD8^+^ T cells, regulatory T cells, and activated memory CD4^+^ T cells, together with increased representation of macrophages M0 and resting CD4^+^ memory T cells. As the risk score increased, immune scores declined, whereas stromal content and tumor purity rose. Overall, higher FMRG scores corresponded to a microenvironment with diminished immune cell presence. Given the central role of folate-dependent metabolism in redox regulation, methylation processes, and nutrient utilization, alterations in these pathways may influence immune cell activity within the tumor setting (18, 23). Folate-driven one-carbon flux contributes to processes that influence T-cell expansion and macrophage polarization, providing a potential link between FMRG activity and immune infiltration patterns. Variation in immune checkpoint expression across risk categories further points to differences in immunotherapy responsiveness associated with folate metabolic status. Together, these observations imply that stratification based on metabolic features could inform therapeutic decision-making in immunotherapy.

Drug sensitivity analysis showed that patients with lower FMRG scores had lower predicted IC_50_ values for cisplatin and gemcitabine, with similar patterns observed for other agents. This suggests that tumors in the high-risk group may be less responsive to chemotherapy.

Mechanistically, folate metabolism contributes to nucleotide synthesis and DNA repair capacity (38). DNA synthesis inhibitors such as 5-fluorouracil and gemcitabine directly interfere with thymidylate synthase and ribonucleotide reductase, enzymes tightly linked to one-carbon metabolism. Furthermore, folate metabolism intersects with mTOR and Wnt signaling pathways, both of which regulate cell survival and therapeutic resistance (14, 39). The broad metabolic influence of folate pathways may therefore account for the consistent association between FMRG_score and response to diverse chemotherapeutic agents, suggesting its utility as a broad indicator for therapeutic grouping.

Among the genes included in the signature, CYP27B1 emerged as a key functional mediator. CYP27B1 encodes 1α-hydroxylase, a critical enzyme in vitamin D metabolism (40). While vitamin D signaling has traditionally been viewed as tumor-suppressive, recent evidence suggests context-dependent roles in cancer biology. Our *in vitro* and *in vivo* experiments demonstrated that CYP27B1 knockdown significantly inhibited proliferation, migration, invasion, and tumor growth while enhancing cisplatin sensitivity. These findings indicate that CYP27B1 contributes to aggressive tumor behavior and chemoresistance in HNSCC. Mechanistically, CYP27B1 may influence tumor progression through modulation of metabolic flux, redox balance, and signaling pathways such as mTOR or Wnt. Given its dual involvement in metabolism and therapy response, CYP27B1 represents a promising therapeutic target within the folate metabolic network.

Collectively, our study emphasizes the central role of folate metabolism in shaping tumor progression, immune microenvironment remodeling, and chemotherapy responsiveness in HNSCC. The FMRG_score can stratify patients by prognosis and may also help identify potential therapeutic targets.

This study has several limitations. The prognostic model was mainly developed and tested using public datasets, and further validation in prospective, multicenter cohorts is needed. Although we confirmed the functional role of CYP27B1, the relationship between folate metabolism and immune regulation still needs further study. Future work should examine whether combining metabolic targeting with immunotherapy or chemotherapy can improve treatment outcomes.

## Data Availability

The original contributions presented in the study are included in the article/Supplementary material, further inquiries can be directed to the corresponding authors.
