# Supplementary material for: Folate metabolism–based risk stratification identifies CYP27B1 as a determinant of tumor progression in HNSCC
Source: Front Med (Lausanne). 2026 May 5;13:1814665. doi: 10.3389/fmed.2026.1814665 (PMC13183860; doi:10.3389/fmed.2026.1814665)
Supplement: Supplementary file 1 [file Table_1.DOCX]

Supplementary Material

# Supplementary Figures and Tables

For more information on Supplementary Material and for details on the different file types accepted, please see [here](https://www.frontiersin.org/guidelines/author-guidelines#supplementary-material).

## Supplementary Figures


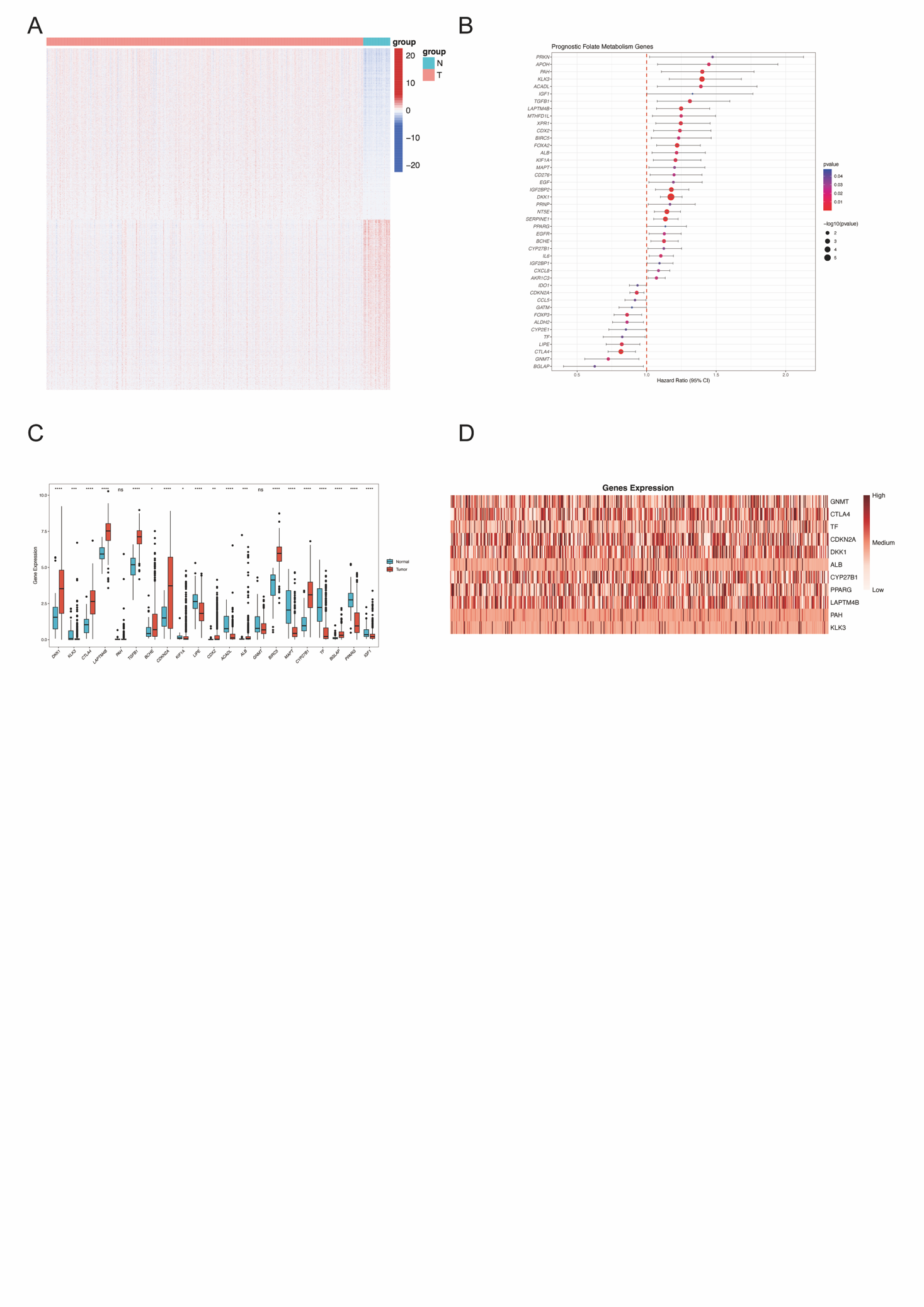


**Supplementary Figure 1. Expression landscape of folate metabolism–related genes in tumor and normal tissues.**

(A) Heatmap showing the expression profiles of folate metabolism–related genes in normal (N) and tumor (T) samples. Samples are annotated by group at the top. Color scale represents normalized gene expression levels (red, high expression; blue, low expression).

(B) Forest plot of univariate Cox regression analysis for prognostic folate metabolism–related genes.

(C) Differential expression analysis of prognostic folate metabolism genes retrieved from lasso regression between normal and tumor tissues. Boxplots display relative gene expression levels. Statistical significance was determined using the Wilcoxon rank-sum test. ns, not significant; *P < 0.05; **P < 0.01; ***P < 0.001; ****P < 0.0001.

(D) Heatmap illustrating the expression patterns of selected key folate metabolism genes across samples.


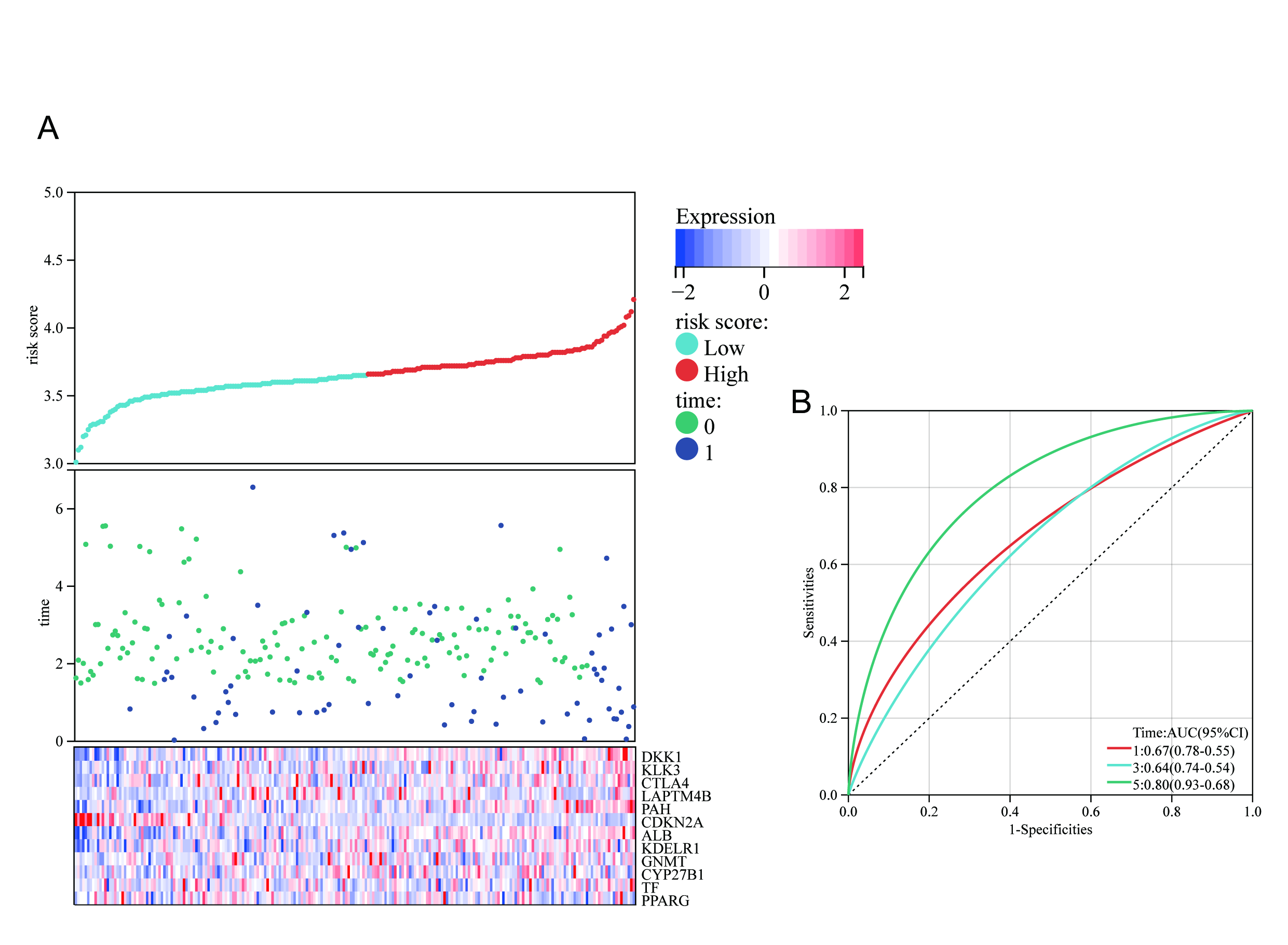


**Supplementary Figure 2. Assessing the performance of the prognostic risk model in the validation cohort.**

(A) Distribution of risk scores, survival status (red dots indicate deceased, blue dots indicate alive), and gene expression of the 11 model genes in the GSE65858 validation cohort. (B) ROC curves for predicting 1-, 3-, and 5-year OS in the GSE65858 validation cohort.


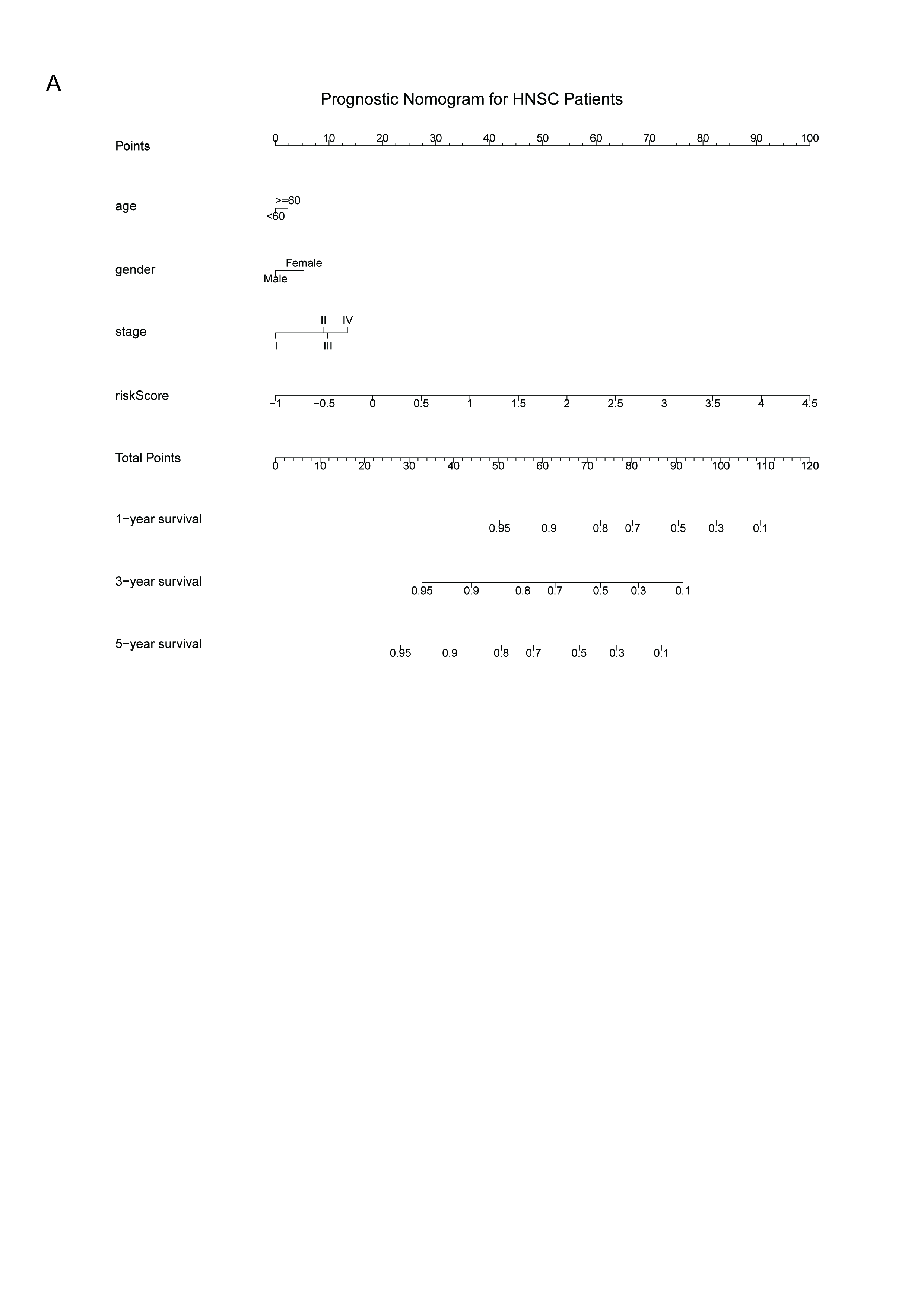


**Supplementary figure 3. Correlation between Clinical Characteristics and the FMRGs related Predictive Signature related to Figure. 2**

(A) Nomogram integrating the FMRG score, T stage, N stage, and age for 1-, 3-, and 5-year 16 OS predictions


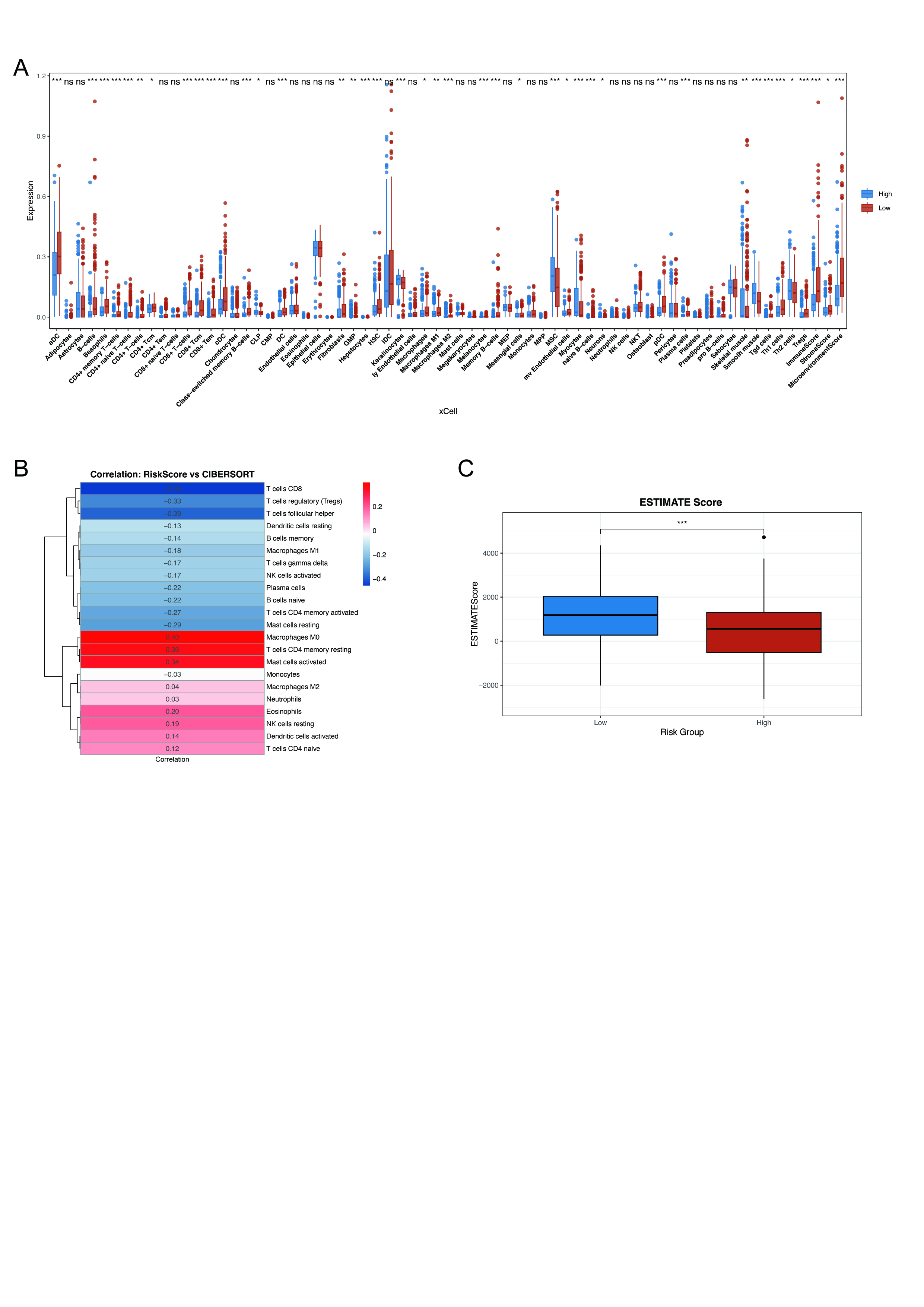


**Supplementary figure 4. Immune landscape and tumor microenvironment characteristics associated with the risk score.**

(A) Comparison of immune cell infiltration levels between low- and high-risk groups based on xCell analysis. Statistical significance was assessed using the Wilcoxon rank-sum test. ns, not significant; *P < 0.05; **P < 0.01; ***P < 0.001.

(B) Correlation analysis between risk score and immune cell fractions estimated by CIBERSORT. The heatmap illustrates Spearman correlation coefficients between risk score and 22 immune cell types. Red indicates positive correlation, and blue indicates negative correlation. Hierarchical clustering was performed to group immune cell types with similar correlation patterns.

(C) Comparison of ESTIMATE scores between low- and high-risk groups. Statistical significance was determined using the Wilcoxon rank-sum test. ***P < 0.001.
